# Paneth-like cells disruption and intestinal dysbiosis in the development of enterocolitis in an iatrogenic rectosigmoid hypoganglionosis rat model

**DOI:** 10.3389/fsurg.2024.1407948

**Published:** 2024-09-09

**Authors:** Iskandar Rahardjo Budianto, Kusmardi Kusmardi, Andi Muh. Maulana, Somasundaram Arumugam, Rejina Afrin, Vivian Soetikno

**Affiliations:** ^1^ Department of Surgery, School of Medicine and Health Sciences, Atma Jaya Catholic University of Indonesia, Jakarta, Indonesia; ^2^ Department of Pathology Anatomy, Faculty of Medicine, Universitas Indonesia, Jakarta, Indonesia; ^3^ Department of Anatomy, Faculty of Medicine, Universitas Muhammadiyah Purwokerto, Purwokerto, Indonesia; ^4^ Doctoral Program in Biomedical Sciences, Faculty of Medicine, Universitas Indonesia, Jakarta, Indonesia; ^5^ Department of Pharmacology and Toxicology, National Institute of Pharmaceutical Education and Research (NIPER) Kolkata, Kolkata, India; ^6^ Department of Pharmacy, East West University, Dhaka, Bangladesh; ^7^ Department of Pharmacology and Therapeutics, Faculty of Medicine, Universitas Indonesia, Jakarta, Indonesia

**Keywords:** Hirschsprung disease, hypoganglionosis, inflammation, antimicrobial peptide, enterocolitis

## Abstract

**Background:**

Hypoganglionosis resembles Hirschsprung disease (HSCR) which is characterized by severe constipation. Enterocolitis due to hypoganglionosis or Hirschsprung-associated enterocolitis (HAEC) is a life-threatening complication of both diseases. This study investigated the role of Paneth-like cells (PLCs) and gut microbiota in the development of enterocolitis in an iatrogenic rectosigmoid hypoganglionosis rat model.

**Methods:**

The rectosigmoid serosa of male Sprague-Dawley rats were exposed to 0.1% benzalkonium chloride (BAC). The rats were then sacrificed after 1, 3, 5, 8, and 12 weeks. A sham group was sacrificed on Week 12. With hematoxylin-eosin staining, the ganglionic cells were quantified, the degree of enterocolitis was analyzed, and the PLCs was identified. Intestinal barrier function was assessed for the anti-peripherin, occludin, and acetylcholinesterase (AChE)/butyrylcholinesterase (BChE) ratio. qRT-PCR was used as reference for the evaluation of antimicrobial peptide (AMP) of PLCs using cryptdins, secretory Phospholipase A_2_, and lysozyme levels. 16S rRNA high-throughput sequencing on fecal samples was performed to analyze the changes in the intestinal microbiota diversity in each group.

**Results:**

After 1 week of intervention, the ganglion cells were fewer in all sacrificial 0.1% BAC groups at varying times than those in the sham group. Occludin and peripherin were decreased, while the AChE/BChE ratio was increased. At Week 5 postintervention, the number of α-defensins-positive PLCs increased in the sigmoid colon tissues from BAC-treated rats. Conversely, PLCs-produced AMP decreased from Week 5 to Week 12. The sham group demonstrated increased *Lactobacillus* and decreased *Bacteroides*, while the 0.1% BAC group exhibited reciprocal changes, indicating dysbiosis. Enterocolitis occurred from Week 1 postintervention.

**Conclusion:**

Application with BAC influences the disruption of PLCs in Week 5 postintervention, and dysbiosis exacerbate the occurrence of enterocolitis. Further research on Paneth cells involvement in HAEC development is warranted.

## Introduction

Hirschsprung disease (HSCR) is a congenital malformation of the enteric nervous system characterized by the absence of ganglion cells (aganglionosis) in the distal colon with the resultant functional obstruction of the gut above the aganglionic segment and spastic contraction of the affected bowel (1). Among the other variants of HSCR is hypoganglionosis, which encompasses enteric neuropathies with a decrease in ganglion cells and has clinical features resembling those of HSCR (2). Hypoganglionosis is distinguished by a decrease in the quantity of parasympathetic nerves in the gut wall and sparse ganglia and is characterized by a very low mucosal activity of acetylcholinesterase (AChE) (3).

Hirschsprung-associated enterocolitis (HAEC) is a severe complication of HSCR that can occur both preoperatively and postoperatively (4, 5). Previous reports have shown that the development of HAEC may be caused by underlying mechanisms such as gut microbiota dysbiosis, bacterial translocation, compromised epithelial barrier function, and gut immune dysfunction (6). Indeed, changes in the gut microbiota in animal and human studies suggest the presence of dysbiosis that precipitates enterocolitis (6, 7). Arnaud et al. demonstrated that hypoganglionosis in large animals disrupts the intestinal barrier, triggering dysbiosis (8). Meanwhile, a previous study showed that in the murine HSCR model, there was an increase in the alpha diversity of the gut microbiota over time compared to wild-type mice (6).

Piebald lethal and lethal spotted strains of mice are the most frequently used animal models to study the pathophysiology of HSCR. Despite the similarities of the animal model of aganglionic genetics to the human HSCR, the aganglionic segment is concise and always located in the distal part of the rectum (9). Moreover, these experimental animals quickly die from severe enterocolitis, which makes it difficult to study the complications of HSCR (10). Sato et al. were able to induce in rats segmental aganglionosis by serosal topical application of benzalkonium chloride (BAC) to the colon and rectum (11). Rat models are easy to use in understanding the pathophysiology and complications of HSCR owing to myenteric plexus ablation and cholinergic nerve fiber hypertrophy resembling megacolon aganglionosis (12). Yu et al., demonstrated that on Days 7–84 of serosal 0.1% BAC-30 min treatment, the number of ganglia and ganglionic cells was subsequently decreased (13).

Paneth cells (PCs) are specialized secretory epithelial cells in the mammalian small intestine that produce various secreted antimicrobial peptides (AMPs) that profoundly influence gut microbiota composition (14). Abnormal appearance of PCs other than small bowel, such as in the distal colon (descending colon, sigmoid, and rectum) are referred to as PCs metaplasia (15, 16). Principally, the functions of PCs are to support the epithelial barrier of the small intestine. This is achieved in two ways, namely (1) PCs help maintain the epithelium's physical barrier by providing nearby intestinal stem cells essential niche signals, and (2) PCs secrete AMPs into the crypt lumen and unstirred intestinal mucus layer (17). Paneth cell function can be disrupted by numerous genetic and environmental factors, including whole-body irradiation, infection, and diet-induced obesity (18). Paneth cell dysfunction compromises AMPs secretion leading to dysbiosis in patients with inflammatory bowel disease (IBD) (15), acute necrotizing pancreatitis (19), necrotizing enterocolitis (NEC) (20), and chronic social defeat stress (21). Pierre et al. demonstrated that before the development of HAEC, transgenic *EdnrB*-null mice with colonic aganglionosis exhibited dysbiosis, impaired mucosal defense, and decreased luminal secretory phospholipase A_2_ (sPLA_2_) (6). However, it remains unclear whether Paneth-like cells (PLCs) in the colon, AMP secretion, and dysbiosis play a role in the development of enterocolitis in rat model of hypoganglionosis. Therefore, to investigate the role of PLCs and their AMP secretion and the changes in the gut microbiota associated with enterocolitis in hypoganglionosis, we used experimental animals treated with BAC at different times over 12 weeks.

## Material and methods

### Animals

All animal testing protocols were approved by Animal Care and Use at Universitas Indonesia following the Universitas Indonesia Regulations of Animal Experimentation (ethical number: 471/UN2.F1/ETIK/PPM.00.02/2022).

Male Sprague-Dawley (SD) rats weighing 150–200 g were purchased from the Indonesian Food and Drug Authority, Indonesia and housed under conventional conditions maintained under a 12 h light/dark cycle with water and food provided *ad libitum*. One cage contained one rat to facilitate the calculation of feces' weight every day.

Thirty-six SD rats were randomly divided into a sham-operated group (sham) (*n* = 6) and a 0.1% BAC group (*n* = 30). The BAC group was further divided into five groups based on the time of termination. Week 1 (W1), Week 3 (W3), Week 5 (W5), Week 8 (W8), and Week 12 (W12) postintervention. To minimize suffering, all rats were anesthetized with 87 mg ketamine/kg and 13 mg xylazine/kg. Subsequently, laparotomy was performed using a 2-cm-long midline incision to identify the sigmoid colon. To prevent bowel dehydration, the paper gauze was wetted with three drops of 0.1% of BAC solution every 5 min. After 30 min of dripping BAC, the paper gauze was released, and the sigmoid colon was washed with 0.9% normal saline. The abdomen was then closed, and all animals were individually housed with a standard diet and tap water *ad libitum*. The sham group rats underwent a sham operation, and the sigmoid colon was washed with 0.9% normal saline for 30 min.

### Abdominal circumference

Abdominal circumference measurements were carried out in all groups of 36 rats, including the sham group (*n* = 6) and the BAC group (*n* = 30). Before sacrifice, abdominal circumference was measured in the largest zone of the rat's abdomen in the ventral position using a plastic non extensible measuring tape with an accuracy of 0.1 cm.

### Postoperative evaluation

All rats were weighed once a week, and their feces were weighed daily. All rats were euthanized by intraperitoneal injection with an overdose of ketamine/xylazine and were sacrificed by exsanguination under deep anesthesia at W1, W3, W5, W8, and W12 after BAC application. The isolated segment of the sigmoid colon was excised from each rat and divided into two parts. One part was promptly fixed in 10% neutral buffered formaldehyde solution for further histological examination, and the other part was washed with cold phosphate-buffered saline (PBS), snap-frozen in liquid nitrogen and stored at −80°C for different experiments. Fresh feces of rats in all groups were collected in sterile tubes and stored at −80°C until further analysis.

### Histologic analysis

For light microscopy observation, the sigmoid colon segment from all rats fixed in 10% neutral buffered formaldehyde solution was dehydrated, embedded in paraffin, and then cut into 5-μm sections. These sections were then stained with hematoxylin-eosin and analyzed by two pathologists blinded to the study. The number of ganglionic cells per ganglia was counted and recorded. Teiltelbaum's scoring was used to analyze the degree of enterocolitis histologically. Grade 0, normal intestinal mucosa; Grade 1, crypt dilation and mucin retention; Grade 2, cryptitis or crypt abscesses; Grade 3, multiple crypt abscesses; Grade 4, crypt hyperplasia and inflammatory cell infiltration; and Grade 5, crypt necrosis (19). Sigmoid colon tissue was used to detect Paneth-like cells based on their eosinophilic granules in the Lieberkühn crypts with hematoxylin-eosin staining using light microscope at a magnification of 1000× (22).

### Immunohistochemistry analysis for α-defensin

For immunohistochemistry (IHC), formalin-fixed, paraffin-embedded sigmoid colon tissue sections were used. After deparaffinization and hydration, the slides were washed in Tris-buffered saline (TBS; 10 mM/L Tris-HCl, 0.85% NaCl, pH 7.2). Endogenous peroxidase activity was quenched by incubating the slides with 0.3% H_2_O_2_ in methanol. The slides were incubated overnight with mouse monoclonal α-defensin 1 antibody (Novusbio, NBP2-75406, Centennial, USA) as the primary antibody, diluted at a ratio of 1:50 at 4°C. Subsequently, the slides were washed in TBS, added with HRP-conjugated recombinant anti-mouse antibody, incubated at 20–22°C for 45 min, then washed again with TBS, incubated with diaminobenzidine tetrahydrochloride as a substrate, and then counterstained with hematoxylin. For semi-quantitative analysis of α-defensin 1, the number of positive cells with strong expression was counted.

### Immunofluorescence analysis for peripherin

Cryosections (5-μm) were cut using a microtome (Bright Instrument Company Ltd., Huntington, UK) housed within a cryostat at −25°C. The sections were collected onto uncoated, precleaned glass slides fixed in 3.7% formaldehyde for 1 h. After rinsing in double distilled water three times for 30 s each, the slides were permeabilized in 0.5% Triton X-100 for 5 min. Subsequently, the slides were washed in PBS (137 mmol/L sodium chloride, 3 mmol/L potassium chloride, 8 mmol/L disodium hydrogen phosphate, and 3 mmol/L potassium dihydrogen phosphate, pH 7.4) for three times 5 min each. The sections were incubated in anti-peripherin antibody (ab4666, Abcam, Cambridge, UK) for 2 h. Following washing in PBS three times for 5 min each, a secondary Alexa Fluor fluorescent-conjugated antibody was applied for 30 min. Secondary antibody controls were included as negative controls. Images were taken with a fluorescence microscope (Olympus, BX43, Japan).

### Gene expression analysis for α-defensin, lysozyme, sPLA2, and interleukin (Il)-1β

Total RNA was extracted from sigmoid colon tissue using a High Pure RNA Isolation kit (Roche Applied Science, Penzberg, Germany) according to the manufacturer's instructions. Nanodrop 1000 Spectrophotometer (Thermo Scientific) was used to measure RNA concentration at a wavelength of 260 nm. To synthesize complementary DNA, 1 μg of RNA, and the Transcriptor First Strand cDNA Synthesis kit (Roche Applied Science) were used according to manufacturer's instructions. Quantitative real-time PCR (qRT-PCR) was conducted using the LightCycler® 480 Instrument (Roche Applied Science) with FastStart Essential DNA Green Master Mix (Roche Life Science). All reactions were performed similarly: 95°C for 10 s, followed by 45 cycles of 95°C for 15 s and 60°C for 1 min. Relative gene expression was calculated using the 2^−ΔΔCT^ method, with β-actin as the housekeeping gene. The following primer pairs were used: for β-actin: Forward: 5′-CTGGTCGTACCACAGGCATT-3′ Reverse: 5′-CTCTTTGATGTCACGCACGA-3′; for α-defensin (cryptdins-1): Forward: 5′-CCG AGA GTG CTT CCT AAA CTA C-3′ Reverse: 5′-AAA GTC TCA GGT GGG ATG TTA G-3′; for lysozyme: Forward: 5′-GAA TGG GAT GTC TGG CTA CTA TG-3′ Reverse: 5′-GTC TCC AGG GTT GTA GTT TCT G-3′; for sPLA2: Forward: 5′-ACT CAT GAC CAC TGC TAC AAT C-3′ Reverse: 5′-GTA TGA GTA CGT GTT GGT GTA GG-3′; and for IL-1β: Forward: 5′-CCA GGA TGA GGA CCC AAG CA-3′ Reverse: 5′-TCC CGA CCA TTG CTG TTT CC-3′.

### Western blotting analysis for AChE, BChE, and occludin antibodies

The frozen sigmoid colonic tissues were weighed and homogenized in an ice-cold Tris buffer (50 mM Tris-HCl, pH 7.4, 200 mM NaCl, 20 mM NaF, 1 mM Na_3_VO_4_, 1 mM 2-mercaptoethanol, 0.01 mg/mL leupeptin, 0.01 mg/ml aprotinin). Homogenates were subsequently centrifuged (3,000× g, 10 min, 4°C), and the supernatants were collected and stored at −80°C. The total protein concentration in each sample was measured by the bicinchoninic acid method. Equal amounts of protein extract (50 μg) were separated by sodium dodecyl sulfate-polyacrylamide gel electrophoresis (Bio-Rad, CA, USA) and electrophoretically transferred to nitrocellulose membranes. Then, the membranes were blocked with 5% skim milk in TBS Tween 20 (20 mM Tris, pH 7.6, 137 mM NaCl and 0.1% Tween 20). Subsequently, the membranes were incubated overnight at 4°C and agitated with primary antibodies against acetylcholinesterase (68 kDa, 1:500, ab97299; Abcam, Cambridge, UK), butyrylcholinesterase (75 kDa, 1:500, AF9024; R&D systems, USA), and occludin (60 kDa, 1:1,000, sc-133256; Santa Cruz, CA, USA). The membranes were washed three times in TBS-T for 15 min and incubated with horseradish peroxidase-conjugated goat anti-rabbit IgG secondary antibody (1:5,000, Santa Cruz) or rabbit anti-mouse IgG secondary antibody (1:5,000, Santa Cruz) for 1 h at room temperature. After a final wash, signals were detected using a chemiluminescence system (ECL western blotting substrate, Life Technologies). To normalize the expression of these antibodies, the protein expression of glyceraldehyde 3-phosphate dehydrogenase (GAPDH) in the same sample was required. Finally, the membranes were scanned and analyzed using ImageJ software.

### Microbiota analysis

Bacterial DNA was extracted from 200 mg rat feces per sample using the ZymoBIOMICS™ DNA Miniprep Kit (catalog #D4300) according to the manufacturer's protocols and then amplified with 341F (CCTAYGGGRBGCASCAG) and 806R (GGACTACNNGGGTATCTAAT) primers, including the V3–V4 region of the bacterial 16S rRNA gene. The PCR products with the proper size were selected by 2% agarose gel electrophoresis. The exact amount of PCR products from each sample was pooled, end-repaired, A-tailed, and ligated with illumine adapters. To generate 250 bp paired-end raw reads, libraries were sequenced on a paired-end Illumina platform. The library was checked with Qubit and real-time PCR for quantification and a bioanalyzer for size distribution detection. Quantified libraries were pooled and sequenced on the Illumina platform according to the required effective library concentration and data amount. Principle component analysis (PCA) was used to analyze the beta diversity, whereas Chao1 index and Shannon index were used to analyze the alpha diversity of gut microbiota among groups. PCA is a statistical procedure to extract principle components and structures in data by using orthogonal transformation and reducing data dimensionalities.

### Amplicon metagenomic sequencing

gDNA samples were amplified with target-specific primer (16SV3-V4). Library preparation was performed using the final PCR products. The final library was sequenced on Illumina platform to generate paired-end raw reads. Adapter and PCR primer sequences from the paired-end reads were removed using Cutadapt. DADA2 was used to correct sequencing errors and remove low-quality sequences and chimera errors. The resulting ASVs data was used for taxonomic classification against SILVA (silva_nr99_v138.1).

### Statistical analysis

Data were analyzed using Prism GraphPad version 9.5.1 software (Boston, MA 02110). Data were reported as the mean ± standard error of the mean. Statistical significance was determined using the one-way ANOVA followed by Tukey's *post hoc* test for multiple comparisons. Differences among groups were considered significant at *p* ≤ 0.05.

## Results

### Body weight, feces weight, and abdominal circumference changes among groups

**Figure 1:**
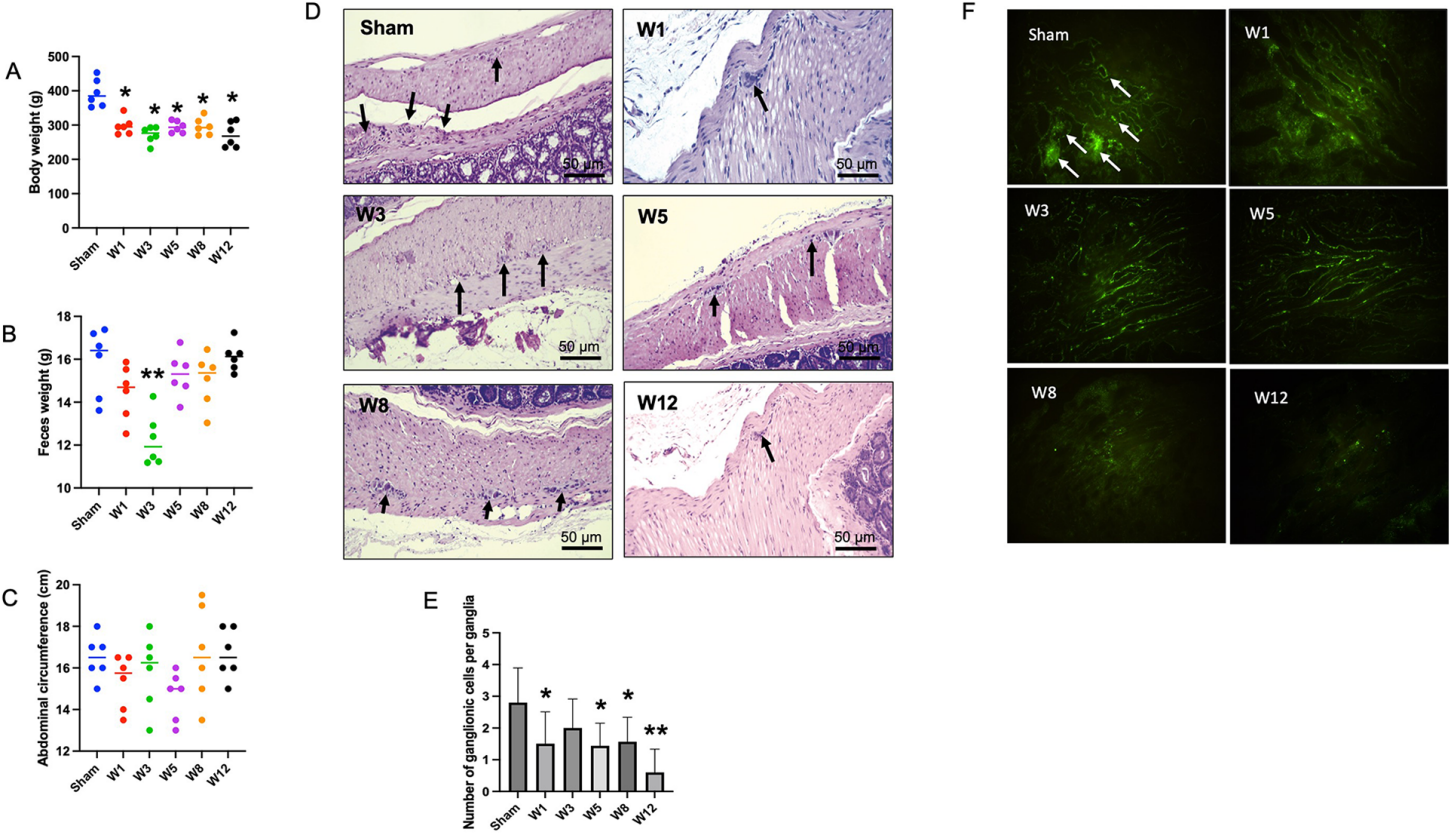
Physical appearance in the sham group and hematoxylin and eosin (H&E) staining of the rectosigmoid colon in the sham group and BAC groups terminated on week 1–12 postintervention. **(A)** Distribution of body weight in each group of rats. The body weight of BAC-treated rats was significantly lower than the sham group (each, *n* = 6). **(B)** Distribution of feces weight in each group of rats. There was no difference in feces weight in BAC-treated rats as compared to the sham group, though feces weight in the W3 postintervention significantly decreased (*p* < 0.01) (each, *n* = 6). **(C)** Distribution of abdomen circumference in each group of rats. There was no difference in the abdominal circumference of BAC-treated rats compared to the sham group. **(D)** H&E staining shows ganglionic cells per ganglia in a 1-cm/5-μm slice of the myenteric plexus. **(E)** Compared to the sham group, the BAC group showed a decreased mean number of ganglionic cells at W1–W12 post-denervation. **(F)** Immunofluorescence staining of peripherin, a type III intermediate filament, showed ganglionic cells in the sham and BAC groups terminated on W1–W12. Magnification 400× for D and F (Scale bar = 50 μm). The data of E was shown as the mean ± SE of six independent experiments in each group. A one-way analysis of variance (ANOVA) test was performed followed by Tukey's test to compare the data. *p ≤ 0.05 vs. sham; **p ≤ 0.01 vs. sham.

All rats survived until the end of the study. At the end of the intervention, the body weight and feces weight were significantly lower in the BAC groups than in the sham group (Figure 1A). However, the difference of feces weight was significant only in W3 BAC group (Figure 1B). Hypoganglionosis caused abdominal distention, which increased according to the time of termination was associated with BAC administration, as supported by the increase in abdominal circumference (Figure 1C).

### Number of ganglionic cells among groups

The number of ganglion cells per ganglia was reduced in the BAC group compared to that in the sham group (Figure 1D). The quantitative analysis showed that the mean number of ganglion cells per ganglia decreased with the time of termination (Figure 1E). Application of BAC successfully resulted in narrow and dilated segments.

All groups had peripherin, an intermediate filament specifically expressed to the peripheral nervous system including enteric ganglion cells. Compared to the sham group, the BAC group showed a decreased expression of peripherin according to the time of termination (Figure 1F).

### Changes in intestinal barrier permeability among groups

**Figure 2:**
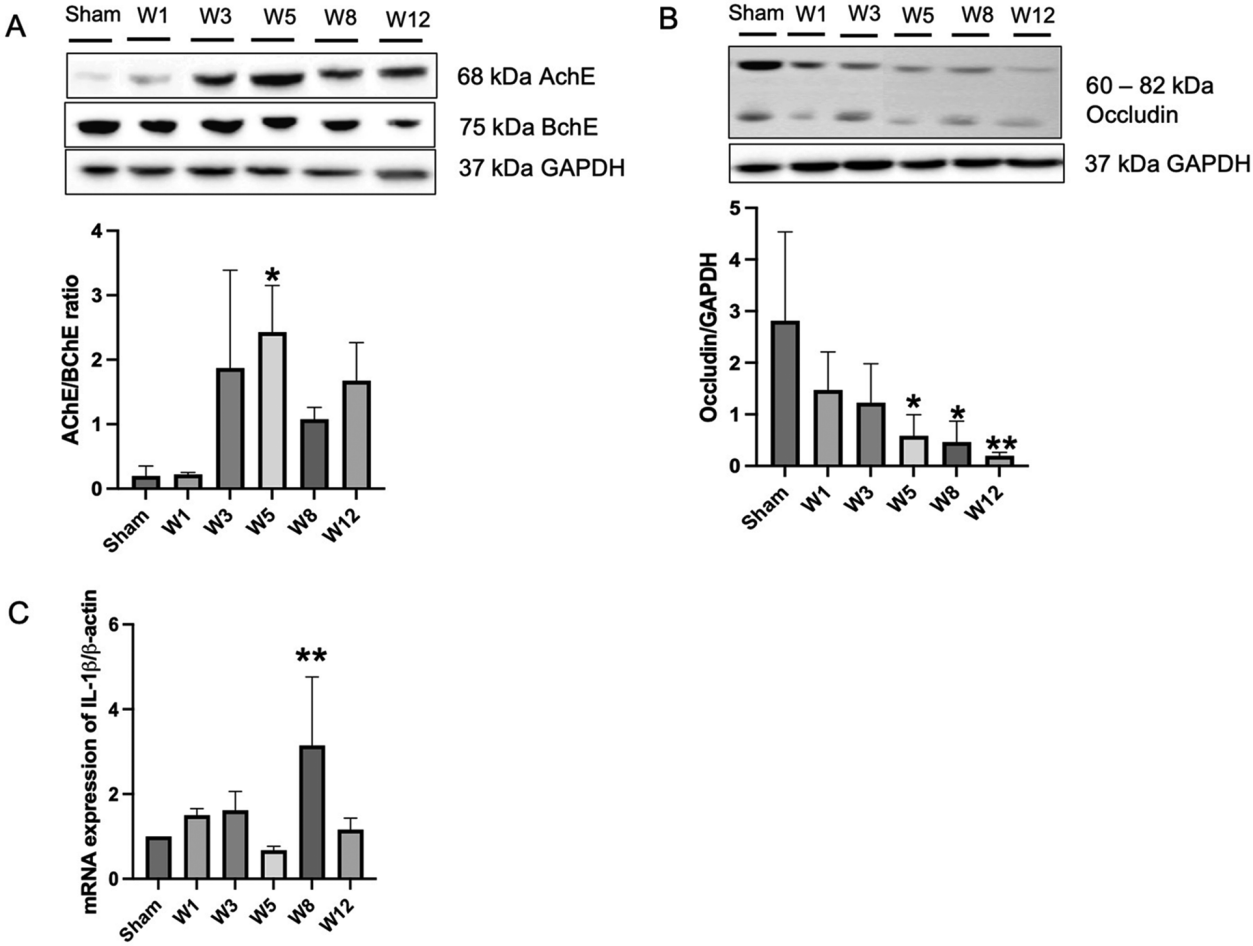
Benzalkonium chloride-induced hypoganglionosis results in hypertrophy of cholinergic nerve fiber, loss of intestinal tight junctions, and inflammation. **(A)** Western blotting revealed an increased AChE/BChE ratio in the rectosigmoid colon of BAC-treated groups (*n* = 6) compared to the sham group (*n* = 6) according to the time of termination. **(B)** Western blotting revealed decreased occludin tight junction in the rectosigmoid colon of BAC-treated groups (*n* = 6) compared to the sham group (*n* = 6) according to the time of termination. **(C)** mRNA expression of IL-1β on rectosigmoid colon tissue between groups. Results are presented as mean ± SE. A one-way ANOVA test followed by Tukey's test was performed to compare the data. *p ≤ 0.05 vs. sham; **p ≤ 0.01 vs. sham.

Next, to evaluate enteric nervous system status, we checked the protein expression of AChE and BChE by Western blotting method. The ratio of AChE/BChE was higher in the BAC groups than in the sham group, mainly at Week 5 postintervention (Figure 2A). This indicates an extrinsic nerve fiber hypertrophy in the sigmoid colon segment. Next, to evaluate tight junction proteins, we examined the expression of occludin protein, one of the tight junction proteins that regulates colonic permeability, in sigmoid colon tissue. Compared to the sham group, the BAC group showed a decreased expression of occludin according to the time of termination (Figure 2B). The functions of the intestinal epithelium are associated with tight junction integrity. To analyze the presence of an inflammatory process due to the loss of intestinal tight junction barrier function, we measured the gene expression of IL-1β, which was increased in the BAC group, mainly at Week 8 postintervention (3-fold) compared to that in the sham group (Figure 2C).

### Modulation of paneth-like cells (PLCs) antimicrobial peptides in BAC-treated rats

**Figure 3:**
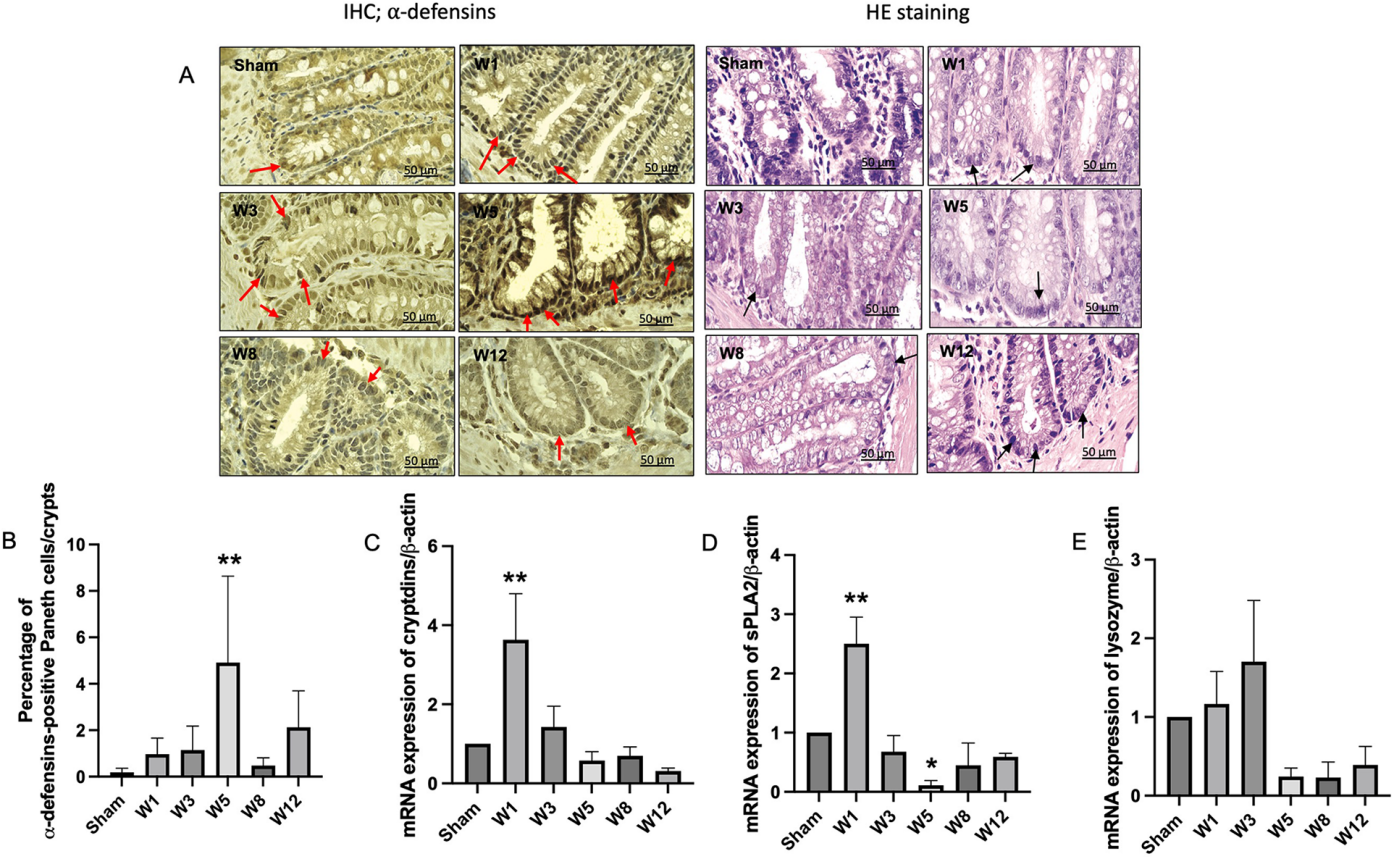
Paneth-like cell metaplasia in the rectosigmoid colon and mRNA expressions of paneth-like cells-secreted antimicrobial peptides. **(A)** Immunohistochemistry for α-defensins-positive Paneth-like cells (PLCs) in the rectosigmoid tissue. Red arrows indicate α-defensins-positive PLCs. PLCs on hematoxylin eosin staining are characterized by dark color, and their location in the crypts of Lieberkühn (black arrows). Magnification 1000× (Scale bar = 50 μm). **(B)** Quantitative analysis of α-defensins-positive PLCs. The sham group showed few PLCs, while the BAC-treated group showed increased PLCs, mainly in W5 in the rectosigmoid colon. The data are shown as the mean ± SE of six independent experiments in each group. A one-way ANOVA test was performed followed by Tukey's test to compare the data. **(C)** mRNA expression of cryptdins, sPLA_2_ **(D)**, and lysozyme **(E)** showed a cycle, increased at W1, indicating PLCs turnover, followed by a decrease until W12, which indicates PLC dysfunction. The data are shown as the mean ± SE of six independent experiments in each group. A one-way ANOVA test was performed followed by Tukey's test to compare the data. **p ≤ 0.01 vs. sham.

On hematoxylin-eosin staining and immunohistochemical analysis of α-defensin to detect PLCs in the sigmoid colon tissue, while the α-defensin-positive PLCs were almost absent in the sham group, these continued to increase in the BAC groups according to the time of termination and peaked at Week 5 postintervention, which proved that PLCs metaplasia occurred in the sigmoid colon tissue of hypoganglionosis rat models (Figures 3A,B). However, no changes were noted in the PLCs in the small intestine adjacent to the colon as they were not mitotic (Supplementary Figures S1, S2).

The gene expression levels of α-defensin (cryptdin), sPLA2, and lysozyme started to increase at Week 1 postintervention and then decreased until the end of the study (Figures 3C–E).

### Composition of luminal microbiota among groups

**Figure 4:**
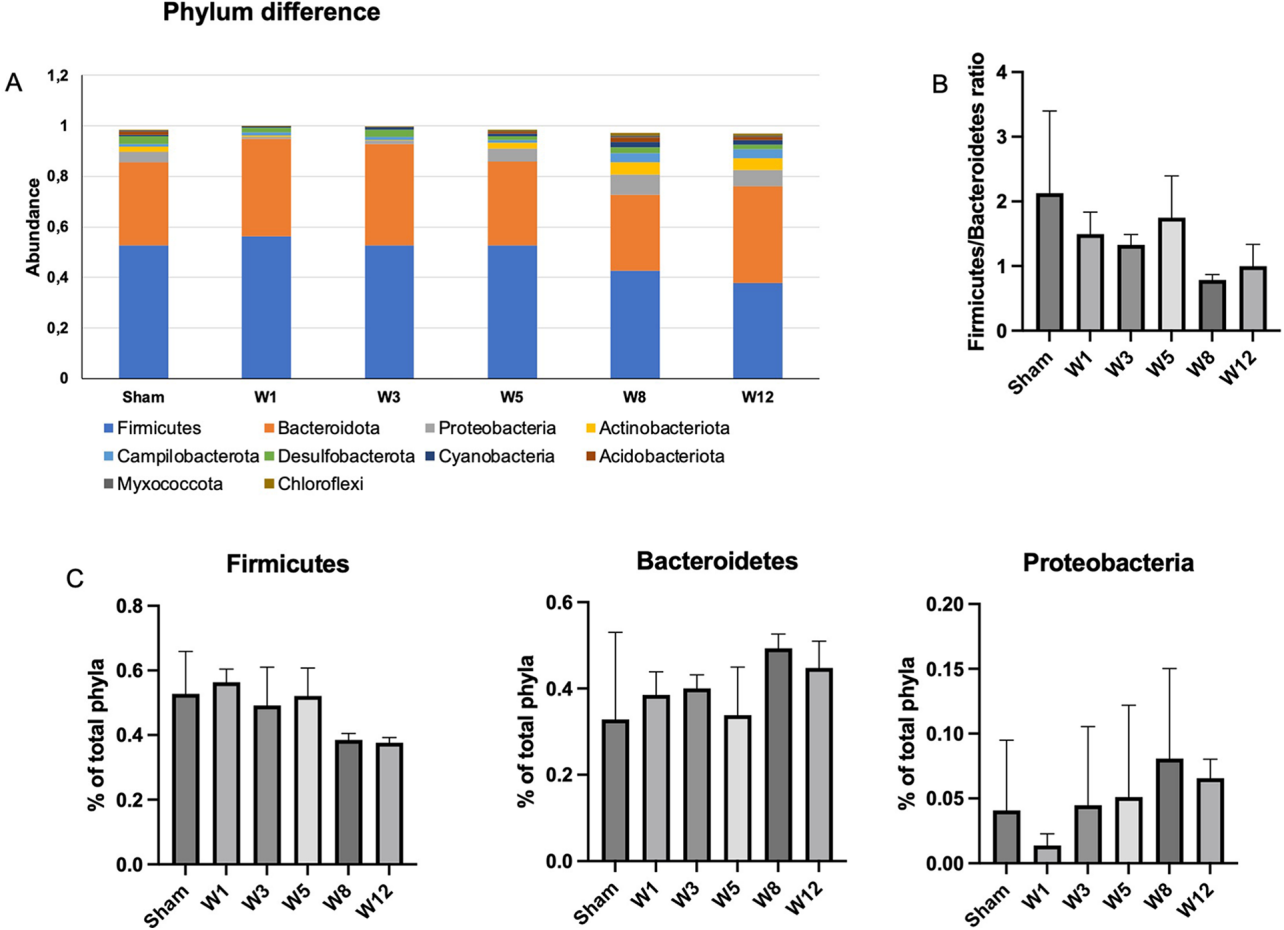
Changes in gut microbiota diversity and structure in hypoganglionosis at the phylum taxonomic. **(A)** Diversity of gut microbiota at phylum levels. **(B)** *Firmicutes/Bacteroidetes* (F/B) ratio among groups was decreased at W8–W12 postintervention. **(C)** Relative abundance of different phyla among groups. *Bacteroidetes* and *Proteobacteria* increased in the BAC groups at W8–W12 postintervention, while *Firmicutes* increased in the sham group. The data are shown as the mean ± SE of four independent experiments in each group. A one-way ANOVA test was performed followed by Tukey's test to compare the data.

The composition of luminal microbiota in the rectosigmoid of the BAC and sham groups was assessed by 16S rDNA sequencing each of four animals. Hierarchical clustering analysis was performed at each taxonomic level from phylum through genus at the time of termination points. At the phylum level, *Firmicutes* and *Bacteroidetes* were the two major bacterial phyla in the gastrointestinal tract of both the sham and the BAC groups (Figure 4A). The *Firmicutes/Bacteroidetes* (F/B) ratio decreased in the BAC group compared to the sham group according to the time of termination (Figure 4B). To further understand the phyla contributing to these grouping, the top three phyla found at each time point of termination were analyzed between groups (Figure 4C). In the sham group, the representation of *Bacteroidetes* and *Proteobacteria* decreased while that of *Firmicutes* increased. Conversely, in the BAC group, *Bacteroidetes* and *Proteobacteria* increased, and *Firmicutes* decreased, mainly at Weeks 8–12 postintervention, but without significant differences between groups (Figure 4C).

**Figure 5:**
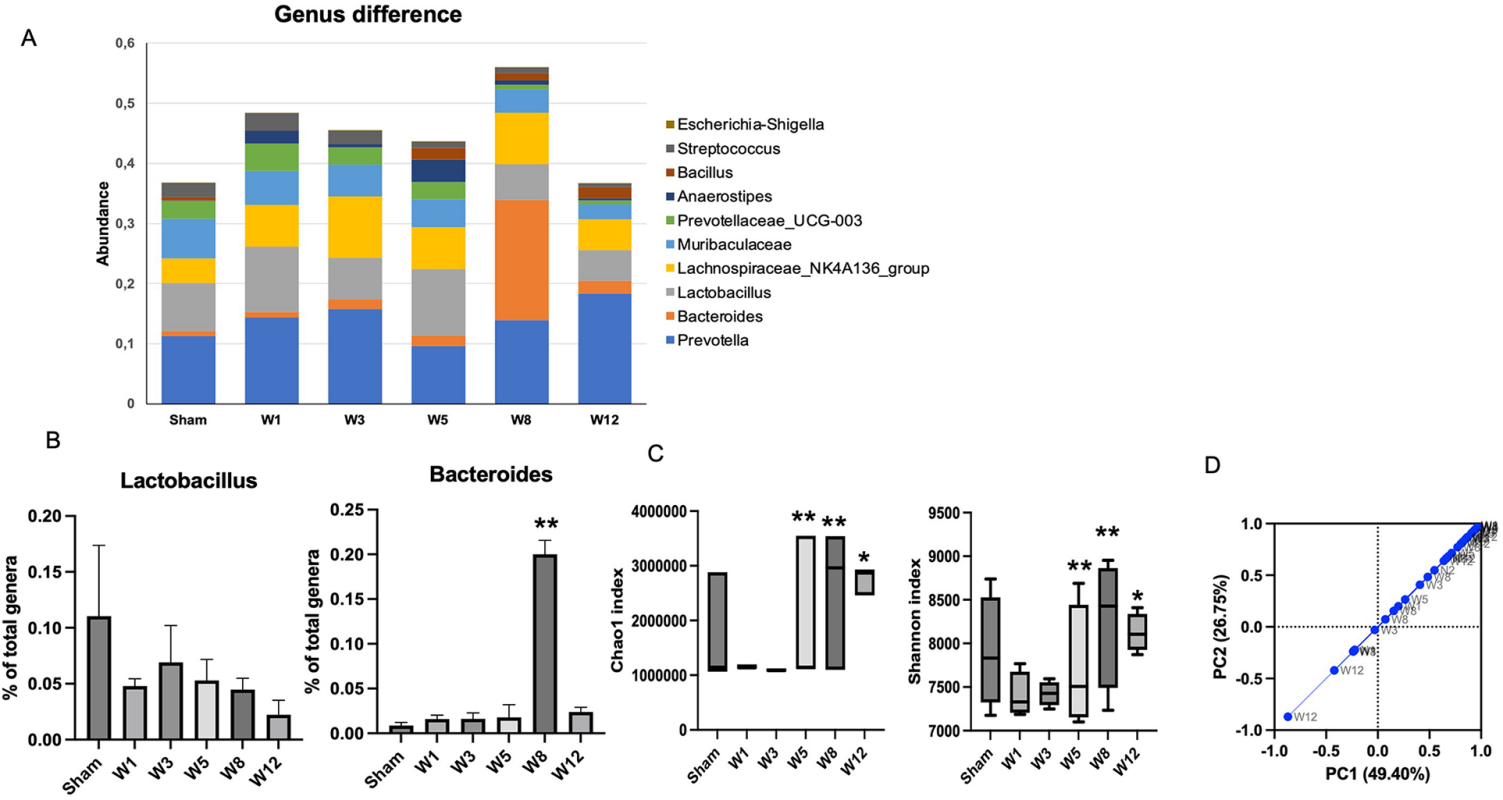
Changes in gut microbiota diversity in hypoganglionosis at the genus taxonomic. **(A)** Diversity of gut microbiota at genus levels. **(B)** *Lactobacillus* increased in the sham group, while *Bacteroides* increased in the BAC groups at W8 postintervention. The data are shown as the mean ± SE of four independent experiments in each group. A one-way ANOVA test was performed followed by Tukey's test to compare the data. **(C)** The richness and diversity significantly differed between early (Week 1[W1] and Week 3 [W3) and late weeks [Weeks 5–12 (W5–W12)]. *p ≤ 0.05 vs. W1–W3; **p ≤ 0.01 vs. W1–W3. **(D)** Difference of beta diversity indices between groups. The composition of microbial communities differed between sham and BAC-treated groups, mainly at Week 12 postintervention.

At the genus level, the top ten genera in each group are shown in Figure 5A. Prevotella is the most common genus found in the sham and BAC groups. *Lactobacillus*, a species considered to provide intestinal mucosal protection, was higher in the sham group and decreased in the BAC group. In contrast, *Bacteroides* increased significantly at Week 8 postintervention compared to the other groups (Figure 5B).

### Microbiota diversity and richness

Richness (Chao1index) and diversity (Shannon index) were similar between the sham group and BAC group (Figure 5C). However, the richness and diversity of microbial communities were significantly different between early weeks (Week 1 and Week 3) and late weeks (Week 5–Week 12) postintervention of BAC. PCA described the β diversity, revealing a separation between the sham group and the BAC group at Weeks 8–12 (Figure 5D). PCA showed that PC1 and PC2 accounted for 49.40% and 26.75% variance, respectively, and PC1 clearly separated the samples in the BAC group in the early and late weeks. This implies differences in the structure of the gut microbiota in early and advanced hypoganglionosis.

**Figure 6:**
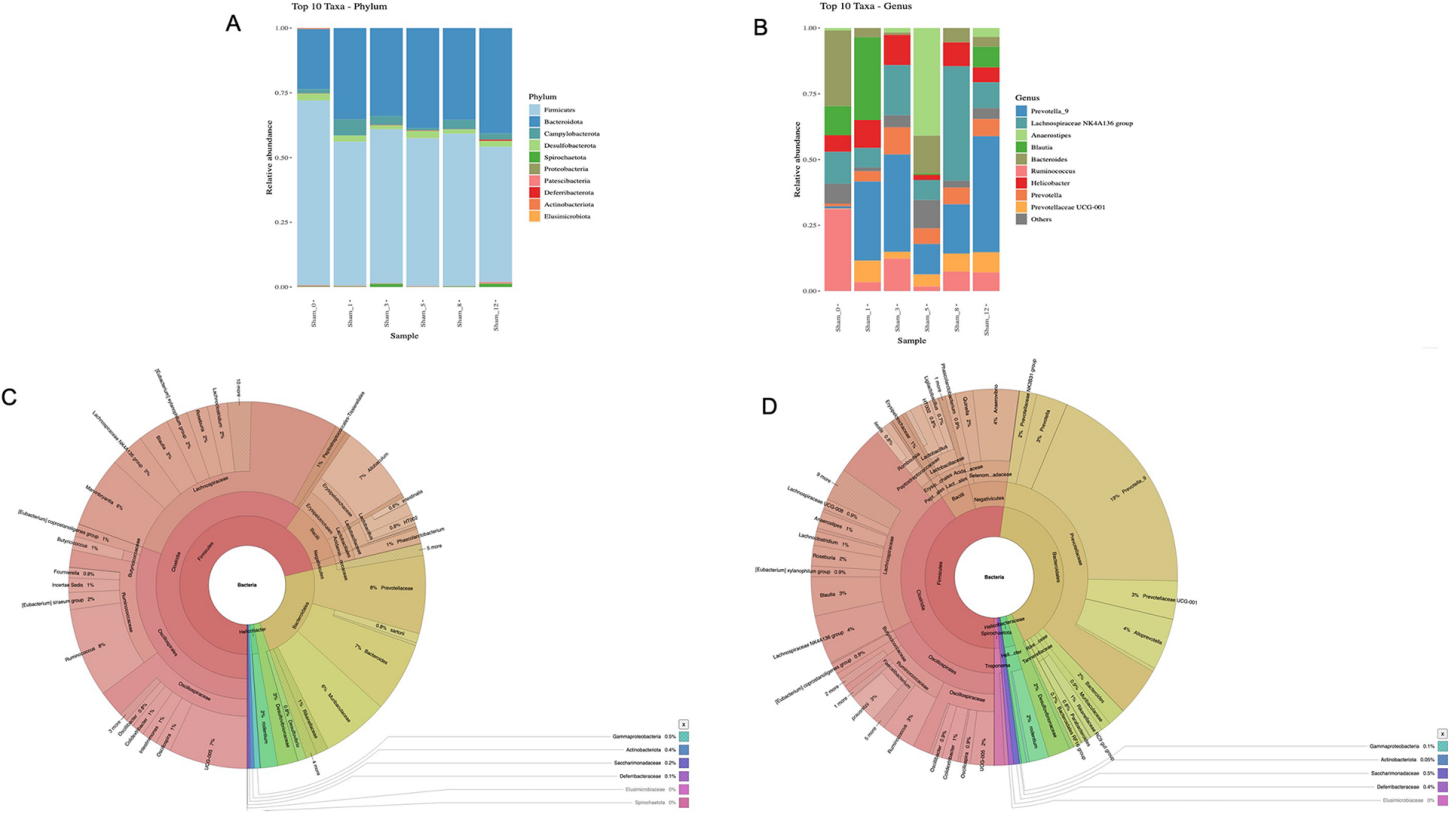
Gut microbiota diversity in the sham group and effect of BAC on microbiota composition. **(A)** Phyla and **(B)** Genus composition of sham group at various times of collection, **(C)** Linear Discriminant Analysis effect size cladogram of sham group terminated at Week 12 and **(D)** BAC group terminated at Week 12 showed that *Bacteroidetes* was the most abundant phyla in the BAC group.

As shown in Figures 6A,B, the levels of abundance of microbiota phyla and genera did not differ in the sham group sacrificed at various time points, while the microbiota diversity at Week 12 was different between the sham group and the BAC-treated group (Figures 6C,D).

### Degree of enterocolitis among groups

**Figure 7:**
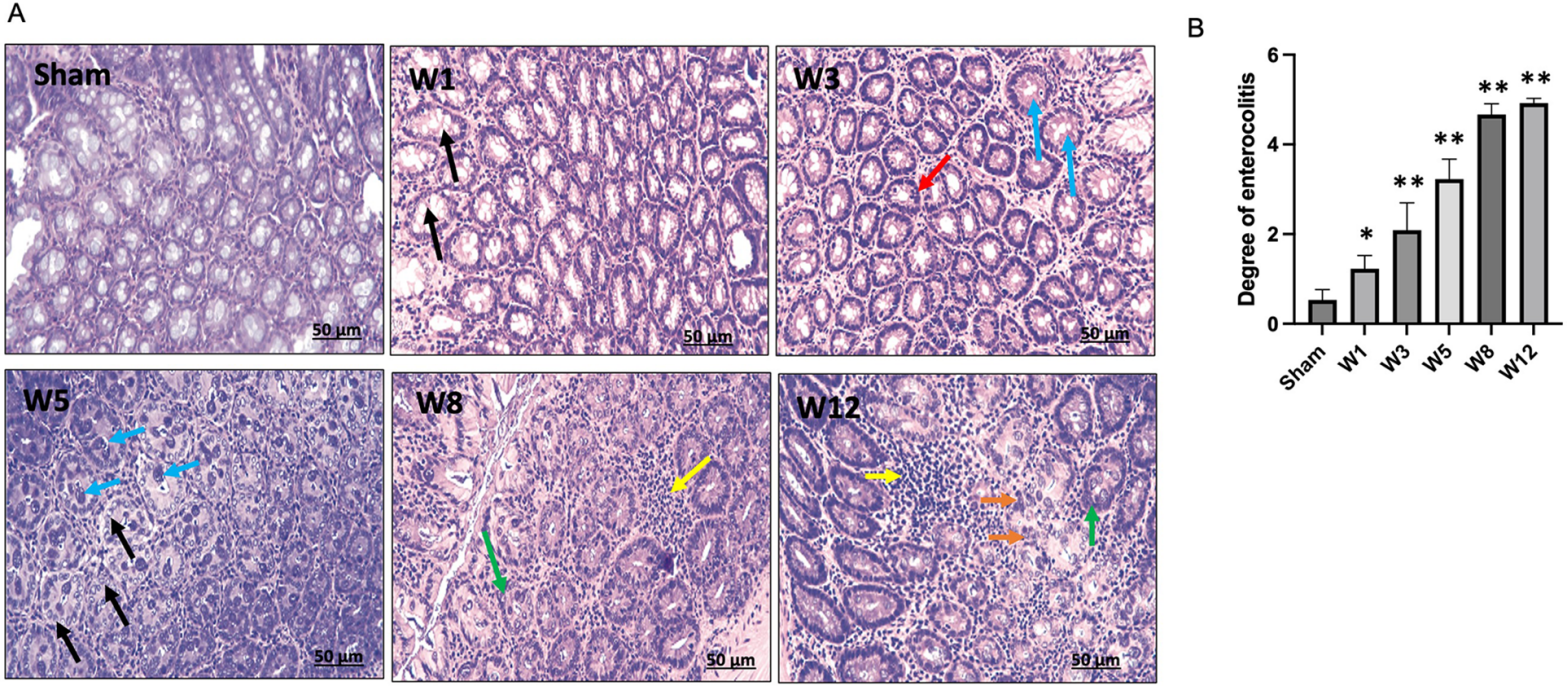
**(A)** Degree of Hirschsprung-associated enterocolitis. [Sham] Grade 0, normal mucosa. [W1] Grade 1, crypt dilation with mucin retention (black arrows). [W3] Grade 2, cryptitis (red arrow) and crypt abscesses (blue arrows). [W5] Grade 3, crypt abscesses (blue arrows) and crypt dilatation with mucin retention (black arrows). [W8] Grade 4, inflammatory cell infiltration (yellow arrow) and crypt hyperplasia (green arrow). [W12] Grade 5, inflammatory cell infiltration (yellow arrow), crypt necrosis (orange arrows), and crypt hyperplasia (red arrow). **(B)** Degree of Hirschsprung-associated enterocolitis between groups. The data are shown as the mean ± SE of six independent experiments in each group. A one-way ANOVA test was performed followed by Tukey's test to compare the data.

The degree of enterocolitis Grade 1–5 was determined in the BAC group (Figure 7A). Grade 1 enterocolitis began to occur in Week 1 postintervention and increased according to the time of termination (Figure 7B).

## Discussion

In the present study, we hypothesized that PLCs and dysbiosis have an important role in the development of enterocolitis in the BAC-induced hypoganglionosis rat model. To induce the hypoganglionosis model in rats, 0.1% BAC was used, which resulted in a decrease in the number of nerve ganglion cells and disruption in intestinal permeability in an exposure time-dependent manner. We showed that the BAC-treated rats not only exhibited abdominal distension and loose feces, especially in the late weeks, and lower body weight, but also experienced enterocolitis, which began in the first week after BAC administration and increased until the Week 12. In addition, the number of PLCs began to increase in the first week, substantially at Week 5, and then decreased until Week 12, as shown by increasing the percentage of α-defensins-positive PLCs without being followed by an increase in AMPs secretion.

In the present study, we used 0.1% BAC concentrations to determine myenteric denervation and hypoganglionosis and showed robust hypoganglionosis at 0.1% BAC. Results of the histopathology analysis showed ablation of the neuronal ganglion cells from the first week and persisting until Week 12 after denervation, as indicated by a decrease in peripherin-positive ganglion cells. Previous studies have demonstrated that peripherin, a type III intermediate filament protein that is specifically exposed in the peripheral nervous system, is a specific neuronal biomarker besides S-100 for diagnosing hypoganglionosis (23, 24). Our findings were consistent with Yu et al., who demonstrated that serosal application of 0.1% BAC to the rat distal colon for 30 min resulted in hypoganglionosis and decreased protein expression of peripherin (13). We also observed that all rats were able to defecate with a soft consistency, despite the development of hypoganglionosis and decreased expression of peripherin. This is most likely because intestinal peristaltic ability still exists. Intestinal peristalsis is stimulated by acetylcholine, the release of which is regulated by stimulation of the intestinal serotonin receptor (25). Nevertheless, additional study is necessary to confirm the modulation of the intestine serotonin receptor resulting from topical BAC administration.

The diagnosis of hypoganglionosis relies on multiple full thickness biopsies of the colon and is characterized histologically by the absence of AChE in the mucosa, reduction of nerve cells in the myenteric and submucosal plexuses, and reduction in the number of ganglion cells (26). Higher AChE activity in HSCR indicates that the AChE/BChE ratio may have discriminatory diagnostic value as the AChE/BChE ratio is markedly decreased in cholinergic nerve fibers in the presence of ganglion cells (27–29). In the present study, the AChE/BChE ratio was higher in the BAC group compared to the sham group, although hypoganglionosis occurred in the BAC group. Further investigation is desired regarding this controversial result.

Occludin is a tight junction necessary to maintain intestinal integrity and acts as a transmembrane signaling protein (30). A reduced expression of occludin in both intestinal epithelial and endothelial cells is associated with increased intestinal barrier permeability and inflammatory process (31, 32). We showed that the protein expression of occludin decreased with time, which was accompanied by an increased secretion of the proinflammatory cytokine IL-1β. Interestingly, the increased secretion of IL-1β was only significant compared to the sham group at Week 8, which then decreased at Week 12. The main proinflammatory cytokine implicated in acute and subacute inflammation that lasts several days to 2–6 weeks is IL-1β, while the proinflammatory cytokines that are mainly secreted in chronic inflammation that lasts several months to years are IL4, IL5, IL6, IL7, and IL13, which might be the cause of this occurrence (33). IL-1β-induced increase intestinal tight junction permeability was mediated by the mitogen-activated protein kinase kinase kinase-1 (MEKK-1)-induced activation of I*κ*B kinase (IKK) catalytic subunits IKKβ and IKKα and activation of NF-*κ*B (34). However, to prove whether the decrease in IL-1β mRNA expression at Week 12 was caused by modulation of MEKK-1 activity, requires further research.

PC metaplasia is characterized by the detection of PCs at other sites such as the stomach and colon under pathological conditions (15, 35). Recently, PCs have been described as hyperplastic and metaplastic in the sigmoid colon of HSCR rats (36). In the present study, PLCs metaplasia occurred in the rectosigmoid colon of the BAC group, and the number greatly increased at Week 5 postintervention, followed by a decrease until Week 12 postintervention. In contrast, very few PLCs numbers could be identified in the sham group (Figure 3B). Our results also showed an increase in gene expression of PLCs secreted AMPs, including lysozyme, sPLA2, and cryptdins at Week 1 postintervention followed by a decrease until Week 12 postintervention. We demonstrated that PLCs became dysfunctional at Week 5 postintervention, whereby PLCs synthesis occurred without the ability to secrete AMPs. Recent investigations have identified numerous diseases that can disrupt normal PC function, resulting in compromised AMP secretion and consequent dysbiosis (37–39). The disruption of PC functions or AMP expression could acutely alter the composition of the cecal microbiome which can ultimately lead to dysbiosis, as was observed before or during NEC development (39). In the present study, Grade 1 enterocolitis characterized by crypt dilation and mucin retention had occurred in the isolated segment of the sigmoid colon at Week 1 postintervention; these results were in agreement with those reported by Cheng et al. in Ednrb mice (40).

The intestinal barrier mediates crosstalk between commensal intestinal microbes and host immunity and is the first line of defense against pathogenic antigens and potentially harmful microorganisms (41). In the present study, the microbial composition of feces from BAC groups was shifted toward opportunistic pathogens such as *Proteobacteria*. Our findings were in line with Li et al., who demonstrated that the most abundant gut microbiota in enterocolitis patients were *Proteobacteria* (42). Previous studies have demonstrated that *Proteobacteria* abundance contributed to dysbiosis and was correlated with a decrease in Firmicutes in IBD patients (43, 44). In this study, we also found that the microbiota abundance varied between the early and late weeks: *Lactobacillus* was the most prevalent microbiota in the early week, whereas *Bacteroides* was the most prevalent microbiota in the late week. Similarly, Pierre et al. proved the similarity of Chao-1 and Shannon's index were similar between the control and EdnrB-null mice groups at an early stage (6). Moreover, the *Firmicutes*/*Bacteroidetes* (F/B) ratio is associated with the maintenance of intestinal homeostasis, and thus changes in this ratio can result in increases in the abundance of *Firmicutes* or *Bacteroidetes* species, leading to obesity and IBD, respectively (45, 46). In our present study, the F/B ratio tended to decrease in the BAC group compared to the sham group.

Enterocolitis-induced shift in the gut microbiota structure was reflected by PCA wherein the composition of microbial communities differed between the sham group and BAC groups, mainly in the late week of enterocolitis, as indicated by α-diversity. There were no differences in microbial abundance and diversity between the sham and BAC groups in the early week. In addition, we showed that the gut microbiota abundance of the sham group that was collected at various time points was the same (Figure 6), which demonstrated that changes in microbiota diversity in the BAC group were caused by hypoganglionosis-induced enterocolitis.

This study has three main limitations. The first limitation is the absence of a sham group at each time of termination due to 3R (Replacement, Reduction, and Refinement) principles regarding animal welfare in our institution. Therefore, the differences in each parameter with the group that received BAC treatment cannot be ascertained. However, in metagenomic analysis of the feces of the sham group terminated at various times, the abundance of gut microbiota was the same at each termination time. The second limitation of our study is lack of electron microscopy to detect PLCs. Instead, we evaluated α-defensins, an AMPs secreted by PLCs in response to stimulation and histopathological examination using hematoxylin eosin staining. The third limitation is that we did not separate the lumen, mucus, and epithelium of sigmoid colon tissue to analyze AMPs secreted by PCs.

**Figure 8:**
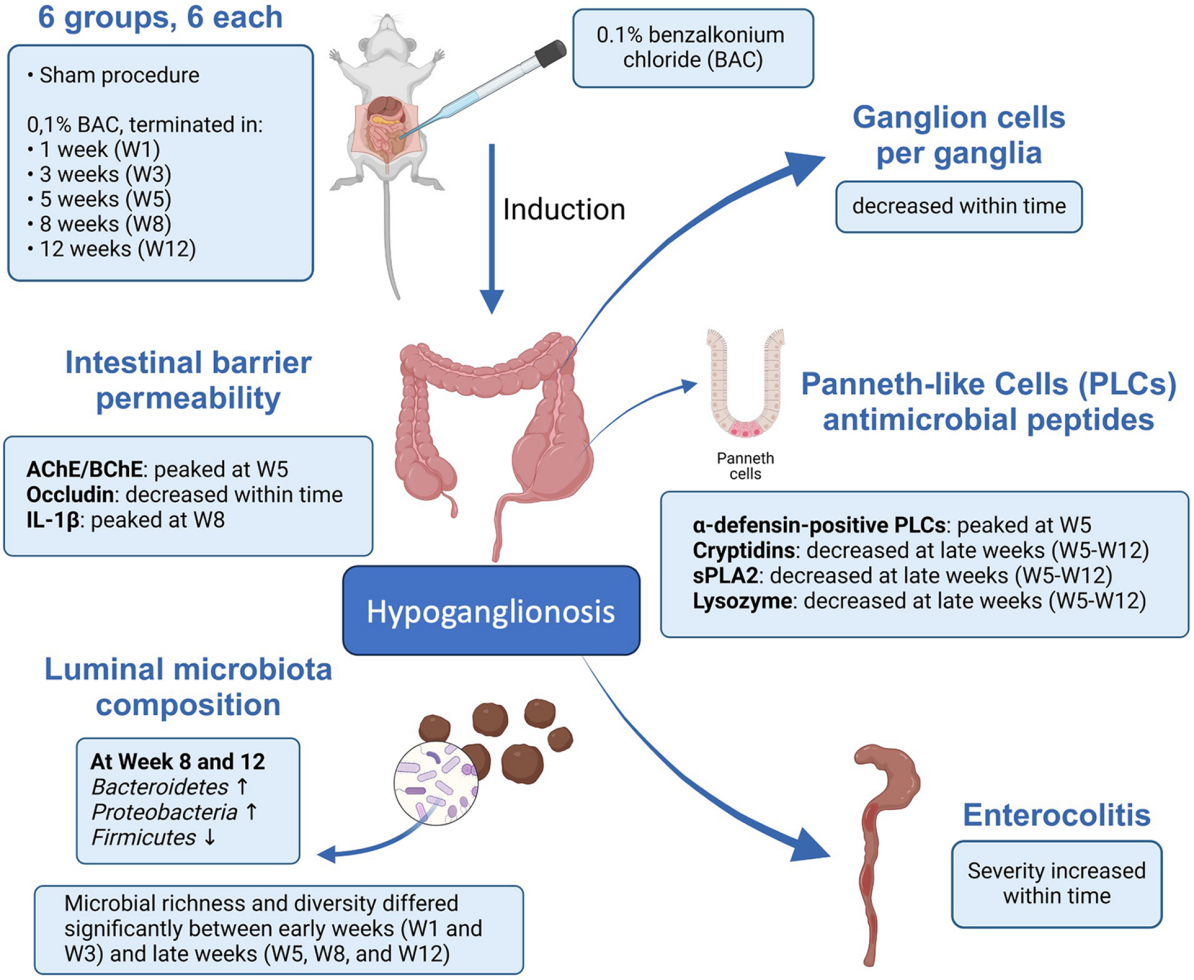
Schematic representation describing the possible mechanism of paneth-like cell disruption induced enterocolitis in an iatrogenic hypoganglionosis rectosigmoid rat model.

In conclusion, the impaired intestinal mucosal barrier and disruption of PLCs functions in maintaining the stability of the intestinal tract are associated with dysbiosis in an iatrogenic rectosigmoid hypoganglionosis rat. This study offers a novel perspective that might be pertinent to the pathogenesis of hypoganglionosis-induced enterocolitis and its proposed mechanisms (Figure 8). Nevertheless, to further support our results, further studies are needed regarding the involvement of PLCs in the development of enterocolitis in hypoganglionosis, with the hope that the management, prevention, and treatment of hypoganglionosis can be enhanced.

## Data Availability

The original contributions presented in the study are included in the article/Supplementary Material, further inquiries can be directed to the corresponding authors.
