# Supplementary figures and images for: Paneth-like cells disruption and intestinal dysbiosis in the development of enterocolitis in an iatrogenic rectosigmoid hypoganglionosis rat model
Source: Front Surg. 2024 Sep 9;11:1407948. doi: 10.3389/fsurg.2024.1407948 (PMC11417098; doi:10.3389/fsurg.2024.1407948)

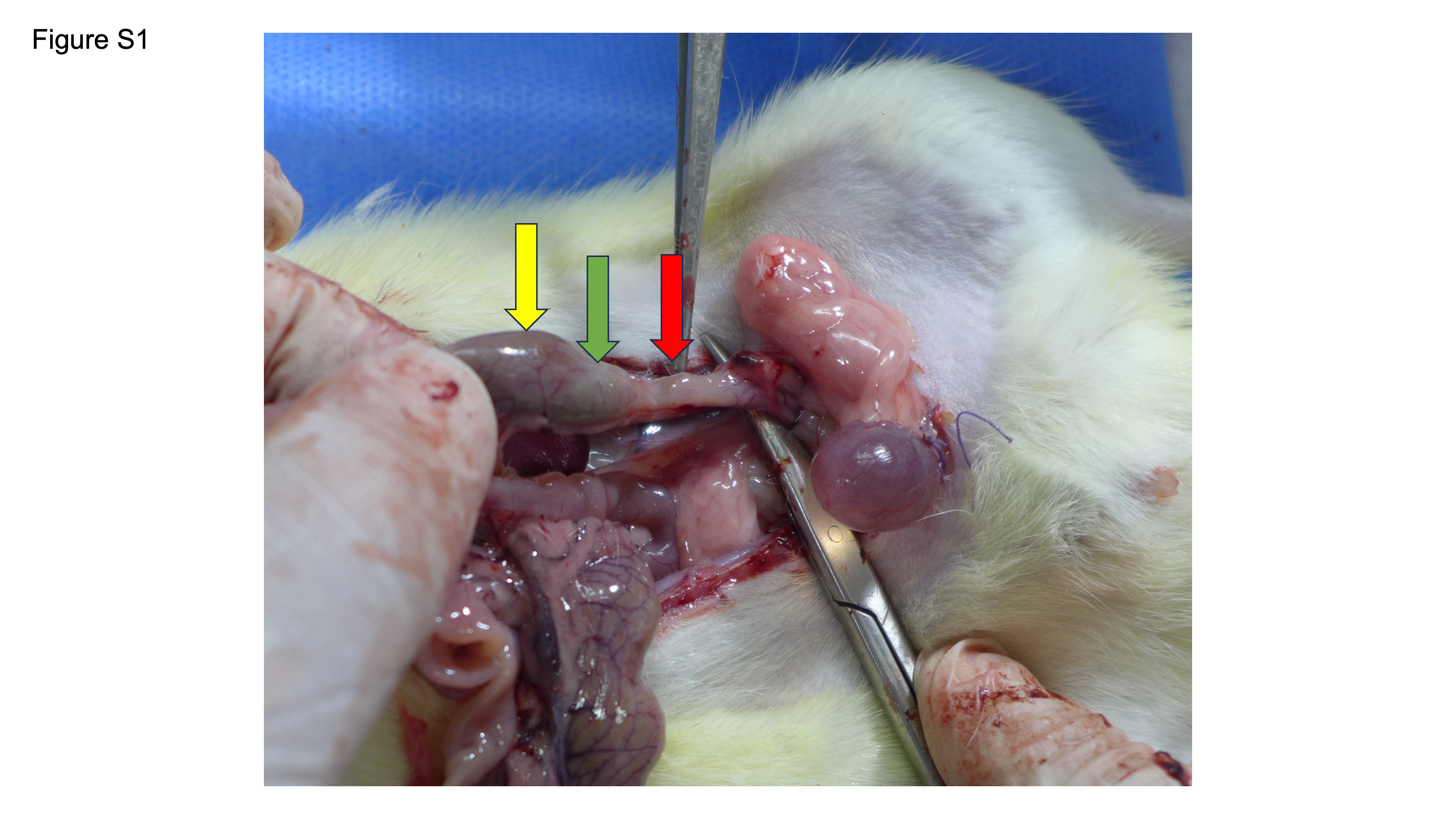

Supplement: Supplementary file 1 [file Image1.tiff]

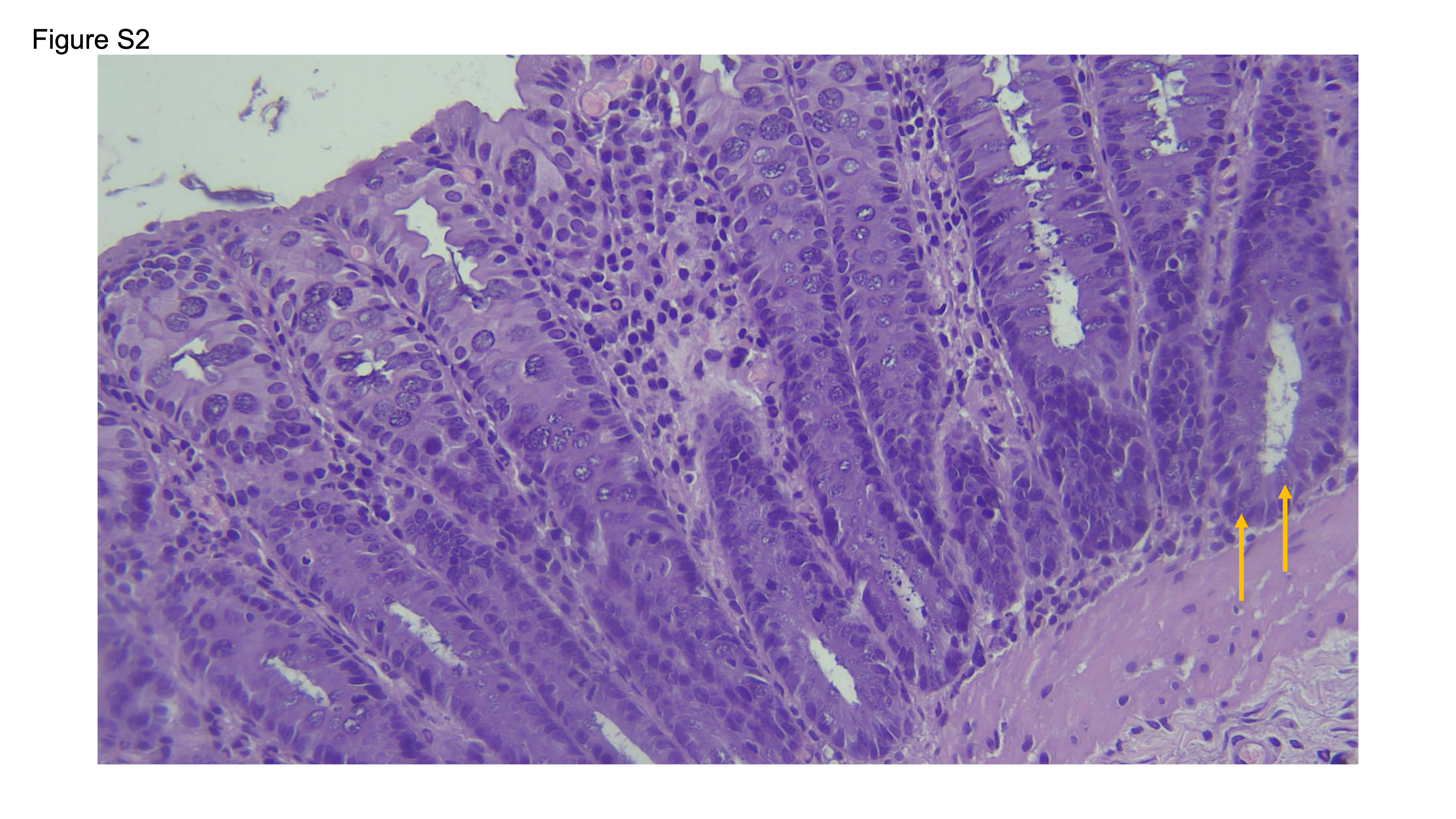

Supplement: Supplementary file 2 [file Image2.tiff]
